# Reducing Disaster Exacerbated Non-Communicable Diseases Through Public Health Infrastructure Resilience: Perspectives of Australian Disaster Service Providers

**DOI:** 10.1371/currents.dis.d142f36b6f5eeca806d95266b20fed1f

**Published:** 2016-12-21

**Authors:** Benjamin J. Ryan, Richard C. Franklin, Frederick M. Burkle Jr., Peter Aitken, Erin Smith, Kerrianne Watt, Peter Leggat

**Affiliations:** College of Public Health, Medical and Veterinary Sciences, James Cook University, Cairns, Queensland, Australia; College of Public Health, Medical and Veterinary Sciences, James Cook University, Cairns, Queensland, Australia; Harvard Humanitarian Initiative, Harvard University, Cambridge, Massachusetts, USA; College of Public Health, Medical and Veterinary Sciences, James Cook University, Townsville, Queensland, Australia; School of Public Health, Queensland University of Technology, Brisbane, Queensland, Australia; School of Medical Sciences, Edith Cowan University, Joondalup, Western Australia, Australia; College of Public Health, Medical and Veterinary Sciences, James Cook University, Townsville, Queensland, Australia; College of Public Health, Medical and Veterinary Sciences, James Cook University, Townsville, Queensland, Australia; College of Public Health, Medical and Veterinary Sciences, James Cook University, Townsville, Queensland, Australia; Faculty of Health Sciences, Flinders University, Adelaide, South Australia, Australia

## Abstract

Background: The exposure of people and infrastructure to flood and storm related disasters across the world is increasing faster than vulnerability is decreasing. For people with non-communicable diseases this presents a significant risk as traditionally the focus of disaster management systems has been on immediate trauma and communicable diseases. This focus must now be expanded to include the management of non-communicable diseases because these conditions are generating the bulk of ill health, disability and premature death around the globe. When public health service infrastructure is destroyed or damaged access to treatment and care is severely jeopardised, resulting in an increased risk of non-communicable disease exacerbation or even death. This research proposes disaster responders, coordinators and government officials are vital assets to mitigate and eventually prevent these problems from being exacerbated during a disaster. This is due to their role in supporting the public health service infrastructure required to maximise treatment and care for people with non-communicable diseases. By focusing on the disaster cycle as a template, and on mitigation and prevention phases in particular, these actions and activities performed by disaster service responders will lead to overall improved preparedness, response, recovery and rehabilitation phases.

Methods: Data were collected via 32 interviews and one focus group (eight participants) between March 2014 and August 2015 (total of 40 participants). The research was conducted in the State of Queensland, Australia, with disaster service providers. The analysis included the phases of: organizing data; data description; data classification; and interpretation.

Results: The research found a relationship between the impact of a disaster on public health service infrastructure, and increased health risks for people with non-communicable diseases. Mitigation strategies were described for all phases of the disaster cycle impacting public health service infrastructure. Specific measures include: increasing the use of telemedicine; preplanning with medical suppliers; effective town planning; health professionals visiting evacuation centers; evacuation centers having power for medical equipment; hubs for treatment and care after a disaster; evacuation of high risk people prior to disaster; mapping people at risk by non-communicable disease; and a mechanism for sharing information between agencies. A common theme from the participants was that having accurate and easily accessible data on people with non-communicable diseases would allow disaster service providers to adequately prepare for and respond to a disaster.

Conclusions: Disaster service providers can play a vital role in reducing the risk of disaster exacerbated non-communicable diseases through public health service infrastructure resilience. They are often employed in communities where disasters occur and are therefore best-placed to lead implementation of the mitigation strategies identified in this research. To sustainably implement the mitigation strategies they will need to become integrated into effective performance and monitoring of the disaster response and health sector during non-disaster periods. For this to occur, the strategies should be integrated into business and strategic plans. Achieving this will help implement the Sendia Framework for Disaster Risk Reduction 2015-2030 and, most importantly, help protect the health of people with non-communicable diseases before, during and after a disaster.

## Introduction

Extreme weather-related disasters are becoming increasingly frequent, due largely to a sustained rise in intensity, severity and frequency of floods and storms (including cyclones, hurricanes, typhoons and tornadoes).^1^^,^ 2 ^,^ 3 ^,^ 4 ^,^ 5 ^,^ 6 ^,^ 7 For example, flooding accounted for 47% of all weather-related disasters from 1995 to 2015, affecting 2.3 billion people, with storms (less frequent) having the highest mortality.^1^ During this period the exposure of the population and infrastructure to weather-related disasters across the world has also increased.^2^^,^ 8

The bulk of ill health, disability and premature death around the globe now generates from non-communicable diseases (NCD).^9^^,^ 10 A combination of population aging, increasing obesity, decreasing physical activity, environmental change and a reduction in communicable diseases has contributed to this epidemiological transition to NCDs.^5^^,^ 6 ^,^ 11 ^,^ 12 ^,^ 13 ^,^ 14 ^,^ 15 However, the traditional focus of the disaster response has been on the management of immediate trauma and communicable diseases.^5^ This focus remains despite the actual risk of post-disaster communicable disease outbreaks being low, particularly in developed countries.^6^^,^ 15 ^,^ 16 ^,^ 17 This highlights the need to refocus disaster risk reduction strategies and resources to the most vulnerable populations.^2^^,^ 3 ^,^ 4 ^,^ 5 ^,^ 6 ^,^ 7 ^,^ 18

The high-burden of NCDs presents a challenge for disaster and health systems, especially when public health service infrastructure (PHI) is destroyed, inaccessible or damaged. In this situation treatment and care is often jeopardized, which can result in disease exacerbation or even death.^5^^,^ 6 ^,^ 14 ^,^ 15 ^,^ 19 ^,^ 20 ^,^ 21 ^,^ 22 ^,^ 23 ^,^ 24 People with NCDs at greatest risk are those with underlying cardiovascular and respiratory diseases, undergoing cancer treatment, unstable diabetes and renal diseases.^6^^,^ 14 ^,^ 20 ^,^ 25 ^,^ 26 ^,^ 27 ^,^ 28 ^,^ 29 PHI priorities in the disaster setting include: workforce; water; sanitation; equipment; communication; physical structure; power; governance; prevention; supplies; service; transport; and surveillance.^30^ All mentioned is vital for maximizing treatment and care for people with NCDs and must be properly maintained.^11^^,^ 31 ^,^ 32

The challenge of NCDs has been recognised in the Sendai Framework for Disaster Risk Reduction: 2015-2030 (Sendai Framework), which is complementary to and building on the World Health Organization (WHO) Global Action Plan for the Prevention and Control of Noncommunicable Diseases – 2013-2020.^10^ NCDs should be part of policy design and implementation of plans to manage risks before, during and after disasters, including having access to life-saving services.^8^^,^ 33

Despite this recognised challenge, research on the strategies required to implement the Sendia Framework and address the impact of disasters on people with NCDs are scarce. This is a significant risk for people in the state of Queensland, Australia. Queensland has recently experienced a number of large scale and devastating natural disasters including cyclones, damaging storms and far reaching floods, and has a high burden of NCDs.^34^^,^ 35 The disasters have included: Cyclone Larry (2006), flash flooding in the Lockyer Valley (2011); and Cyclone Yasi (2011).^16^ Also, NCDs cause approximately 90% of all deaths, account for 88% of the burden of disease and are responsible for 83% of recurrent health expenditure in Queensland.^36^^,^ 37 The burden of NCDs, although lower at 78%, is also a major concern for Indigenous people with these conditions being responsible for 80% of the mortality gap (69.1 years for males, 10.6 years lower than non-Indigenous; 73.7 years for females, 9.5 years lower than non-Indigenous).^38^^,^ 39 The threat of natural disasters for vulnerable populations with NCDs is expected to continue and is anticipated to increase, with climate change expected to make extreme weather events such as cyclones and floods more frequent.^34^^,^ 40

This paper is part of a project aiming to develop strategies for reducing disaster exacerbated NCDs through PHI resilience.^5^^,^ 6 ^,^ 15 ^,^ 30 Specifically this study focuses on the perspective of responders, coordinators and government officials designated as disaster service providers. In the wider project different approaches have been used to gather information around NCDs and PHI resilience. Participant groupings include: environmental health professionals (via focus groups)^15^; disaster service providers (via interviews and a focus group); and people with NCDs (via interviews). Combining these groups into one research paper would have greatly missed the individual challenges, nuances and recommendations being understood and placed on each group. Each group has a unique, discrete and important perspective, for example, the environmental health profession addresses risks relate to drinking water, sanitation, food safety and mass gatherings.^32^^,^ 41 Disaster service providers are responsible for overall leadership, designing and managing system-wide preparation, prevention, response and recovery phases of disaster management.^42^ Meanwhile, people with NCDs represent the group at greatest risk of disease exacerbation if impacted by a disaster.^6^

The aim of this research was to explore the role of disaster service providers in reducing disaster exacerbated NCDs through PHI resilience. To achieve this, interviews and a focus group were conducted with disaster service providers in the State of Queensland, Australia. This included: investigating descriptions of PHI from their perspective; the impact of disasters on PHI and proposed resilience strategies; disaster impact by PHI and NCD; and identification of mitigation strategies by PHI.

## Methods

Data was collected via 32 interviews and one focus group (eight participants) between March 2014 and August 2015 with 40 disaster service providers representing ten organizations. The qualitative analysis included the phases of: organizing data; data description; and data classification and interpretation.^43^^,^ 44 ^,^ 45 A description of the research area, definitions and the analysis process are provided in the following.

**Research area**

The research areas included: Cairns and Hinterland Hospital and Health Service (HHS); Darling Downs HHS; and Townsville HHS. All are located in the State of Queensland, Australia. Government officials with disaster management responsibilities in Brisbane (State Capital) were also included as their decisions directly influence PHI, management of NCDs and disaster responses.

The focus on Cairns and Hinterland HHS, Darling Downs HHS and Townsville HHSs was due to their NCD burden and recent disasters.^46^^,^ 47 For example, Cyclone Larry (2006), flash flooding in the Lockyer Valley (2011); and Cyclone Yasi (2011), which resulted in widespread damage to PHI.^16^ Natural disasters are a feature of the climate and landscape in these regions and are anticipated to increase, with climate change expected to make extreme weather events such as floods and storms more frequent.^16^^,^ 34 ^,^ 40

The Cairns and Hinterland HHS services a population of just over 280,000. Of this population, 9% are Indigenous, compared to 3.5% for Queensland and 2.5% for Australia.^48^^,^ 49 The Darling Downs HHS services a population just under 280,000 and Indigenous people make up 4.2% of the population.^50^ The Townsville HHS services a population of approximately 240,000 and 7% are Indigenous.^51^ There are no demographics for Brisbane because this is primarily the location of government officials who influence PHI, management of NCDs and disaster responses from a state-wide perspective.

**Definitions**

*Disaster service providers*

The experiences of three types of disaster service providers were explored: responders, coordinators and government officials. These disaster service providers were invited to participate because their perceptions, experience and knowledge is vital in influencing strategies for reducing disaster exacerbated NCDs through PHI resilience. A description of each group is provided below:

- Responder: This group includes doctors, nurses, paramedics, police officers and representatives from non-government organizations such as the Australian Diabetes Educators Association, Australian Red Cross and the Kidney Support Network. Discussions with this group addressed the preparedness, prevention, response and recovery arrangements and the role of various individuals, organizations and leading local players in disaster health management.^42^

- Coordinator: This group includes local disaster coordinators, members of local and district disaster management groups and disaster management officers from Queensland Fire and Emergency Services. This group provides a conduit between Local and State Disaster Management Group’s and has specialist knowledge of the principles of disaster management.^42^

- Government officials: This group includes representatives from the Department of Health (Queensland), Queensland Police Service, Queensland Fire and Emergency Services and Queensland Ambulance Service with a state-wide disaster management role. This group was based in Brisbane, the State Capital of Queensland, Australia. Overall this group is responsible for leading, designing and managing the state-wide preparation, prevention, response and recovery phases of disaster management.^42^

*Non-communicable disease (NCD)*

The NCD terms used to guide the analysis were cancer, cardiovascular diseases, diabetes, respiratory conditions and renal diseases.^6^ These conditions are at greatest risk of exacerbation after a disaster and are long-lasting and place great burden on patients, health services and fiscal systems.^47^^,^ 52 A category of NCD-general was used for additional conditions identified by participants.

*Public health service infrastructure (PHI)*

PHI and the associated services were considered the workforce, equipment, supplies and services required to maintain the health and well-being of individuals and the community.^30^ Beyond this definition, there were 13 PHI themes used to guide the analysis and (in priority order) included: workforce; water; sanitation; equipment; communication; physical structure; power; governance; prevention; supplies; service; transport; and surveillance.^30^

*Resilience*

Resilience is the capacity to prevent, mitigate, prepare for, respond to, and recover from the impacts of disasters.^53^^,^ 54 In this context resilience focuses on enhancing the ability of PHI to minimize the effects of future disaster events on people with NCDs. Resilience is a dynamic quality and is usually developed and strengthened over time, it builds upon rather than replaces existing strengths and arrangements.^54^

**Data collection**

Data were collected through interviews and focus groups, which were guided by a combination of structured and open-ended questions developed by the research team. The focus group acted as a group interview where the same questions were asked, however, the answers were provided in an open discussion. The professions targeted by the interviews and focus group were similar.

The aim of the data collection phase was to understand the role disaster service providers could have in maximizing the treatment and care for people with NCDs during and after a disaster. The questions focused on participant’s examples of PHI; relationship between a breakdown of PHI, and poor health outcomes for people with NCDs; and mitigation strategies.

The participants were recruited and selected using a purposive samplingTable strategy.^43^ A focus group was held in Townsville and interviews conducted in Brisbane, Cairns and Innisfail (Cairns and Hinterland HHS), Townsville (Townsville HHS) and Toowoomba (Darling Downs HHS).

The principle of saturation was used to determine the number of interviews.^43^^,^ 44 ^,^ 55 ^,^ 56 For convenience a focus group was held in Townsville as part of a regional disaster meeting. The point of saturation was achieved after approximately 25 interviews. The focus group and remaining six interviews did not generate any new information. The additional interviews were held to ensure all organizations and research locations had an opportunity to participate.

**Data Analysis**

A deductive content analysis was used to analyse the data for both the interviews and focus groups. This included the phases of: organizing data; data description; and data classification and interpretation.^43^^,^ 44 ^,^ 45 The process is described in the following:

1. Organizing data: the focus group and interviews were tape recorded and then transcribed. After transcription the data was saved electronically and then uploaded to QSR NVivo 10.
2. Data description: A description of the data was developed based on the key phrases, ideas and concepts. Interview participants were allocated a de-identified code, for example, *I1*, and focus group data was referred to as *FG*. Firstly, ‘lean coding’ was used to analyse the data. The terms were based on the descriptions of PHI, and NCDs; mitigation strategies; and negative, positive and neutral impact of flood and storm related disasters on PHI, and NCDs. Secondly, the codes were placed in a Microsoft Excel™ spreadsheet to develop an individual description based on the key phrases, ideas and concepts.
3. Data classification and interpretation: The data was classified through an aggregation of individual descriptions and themes in a Microsoft Excel™ spreadsheet to create an overall case description. The data was analyzed by comparing PHI descriptors with the literature, describing NCD descriptors, outlining the impact of disasters on PHI and resilience strategies, reporting the impact by PHI and NCD, and demonstrating mitigation strategies by PHI theme. The data was then interpreted in the discussion to understand the role of disaster service providers in mitigating the impact of disasters on treatment and care for people with NCDs.

**Ethics**

Ethics approval was provided by James Cook University (H4871), Australia, and Townsville Hospital and Health Service Human Research Ethics Committee (HREC/13/QTHS/251). Site specific ethics approval was received from the Cairns and Hinterland HHS (SSA/15/QCH/54 – Lead 147), Darling Downs HHS (SSA/14/QTDD/21) and the Townsville HHS (SSA/14/QTHS/153). This was complemented by letters to specific agencies and disaster management groups seeking permission to invite staff to participate in the research.

## Results

**Participants**

The majority of the 40 participants were responders (n=16) followed by coordinators (n=14) and government officials (n=10) (Table 1). There were 17 female and 23 male participants. The majority of participants were from agencies in Brisbane (n=15) followed by the Townsville HHS (n=13), Cairns and Hinterland HHS (n=8) and Darling Downs HHS (n=4). Of the Brisbane based participants, five were categorized as responders as they worked for non-government agencies.

**Figure 1:**
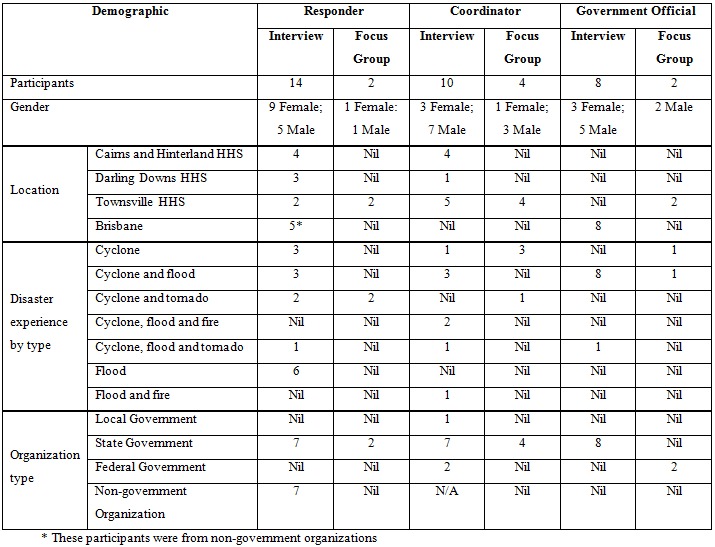
**Table 1.** Demographics

All participants had responded to a natural disaster and were in some way involved in disaster management. Most had experienced multiple disaster types with cyclone and flood the most common (n=15) followed by cyclone and tornado (n=5); cyclone, flood and fire (n=2); cyclone, flood and tornado (n=3); and flood and fire (n=1). The most common single type of disaster experience was cyclone (n=8) followed by a flood (n=6).

The majority of participants were from State Government agencies (n=30). The majority of this group were from the Department of Health and HHSs (n=16) followed by the Queensland Police Service (n=9), Queensland Fire and Emergency Service (n=3) and the Queensland Ambulance Service (n=2). Other participants were from non-government organizations (n=7), Australian Government (n=2) and Local Government (n=1).

**Public health service infrastructure (PHI) descriptors**

The interviews and focus group identified 130 different descriptors of PHI, which were grouped into 14 themes (Table 2). In comparison, Ryan et al^30^ identified 167 different descriptors for PHI, which were grouped into 13 themes. The disaster service providers identified more descriptors for prevention and water. Meanwhile, the literature identified more descriptors for all other themes. An additional theme of ‘other’ was identified by the participants. This was used to group pets which according to the participants should be considered a descriptor for PHI. Additional descriptors were identified by the participants for all thematical areas except power. The data is provided in Table 2.

**Figure 2:**
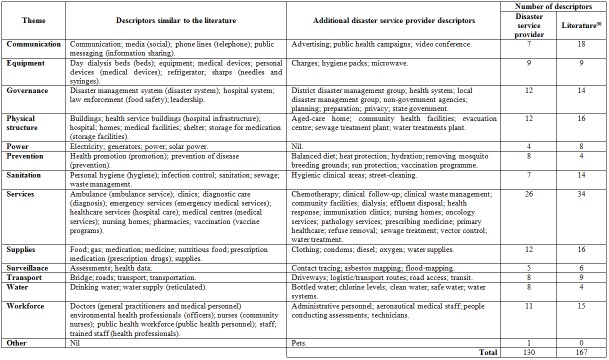
**Table 2.** Descriptions of public health service infrastructure - disaster service provider perspective

**Disasters impact on public health service infrastructure and proposed resilience**

The participants described various impacts of disasters on PHI (n=56) and proposed resilience strategies (n=37), which were categorized into two descriptors (reported impact and proposed resilience strategy). The descriptions were categorized into PHI themes outlined in Table 3 and are discussed below.

**Figure 3:**
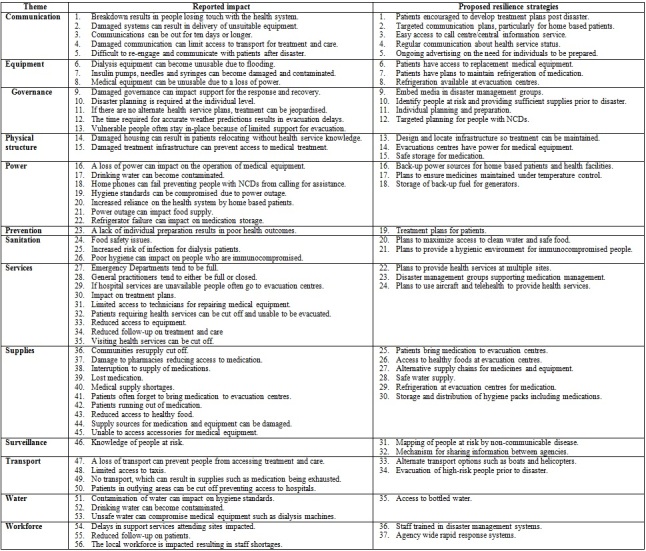
**Table 3.** Disaster impact on public health service infrastructure and proposed resilience strategies

*Impact on public health service infrastructure (PHI)*

The participants reported flood and storm related disasters can impact all aspects of PHI. Damage to communication, equipment, power, services and supplies can result in people losing touch with the health system, limiting access to treatment and patients running out of medications. Governance, transport and workforce is often interrupted or overwhelmed, which can impact on the response and recovery, reduce access to treatment and result in some vulnerable people not being evacuated due to a reduction in support resources. Damage to physical structure, sanitation and water can prevent access to treatment, compromise medical equipment, result in poor hygiene standards and increase the risk of infection. An interruption of surveillance reduces the knowledge-base about people at risk, making it difficult to properly target resources. Post disaster, any inadequacies in prevention activities such as poor town planning, inadequate stockpiling/storage of medications and lack of individual preparedness by with people with NCDs becomes evident. The result is interrupted treatment and care and an increased risk of poor health outcomes for people with NCDs.

*Resilience strategies*

The participants described resilience strategies for all phases of the disaster cycle impacting on PHI. For communication, equipment, services and supplies this included encouraging targeted communication, use of telehealth, patients having their own treatment plans post disaster, providing access to back-up medical equipment and plans to provide health services at alternate sites. Governance, transport, workforce and surveillance strategies could include embedding media in disaster management groups, targeted planning for people with NCDs, use of alternate transport options, early evacuation and agency-wide staff training. Physical infrastructure and sanitation could involve designing and locating infrastructure to allow treatment to be maintained and plans to maintain a safe water supply. Resilience for power could include evacuation centers having the capability to power medical equipment and back-up power sources for home based patients and health facilities. Water supply could become resilient by ensuring access to bottled water.

**Impact of disaster by public health service infrastructure and non-communicable disease**

The participants reported relationships between the impact of a disaster on PHI, and increased health risks for people with NCDs. These relationships are outlined in Table 4 and discussed in the following.

**Figure 4:**
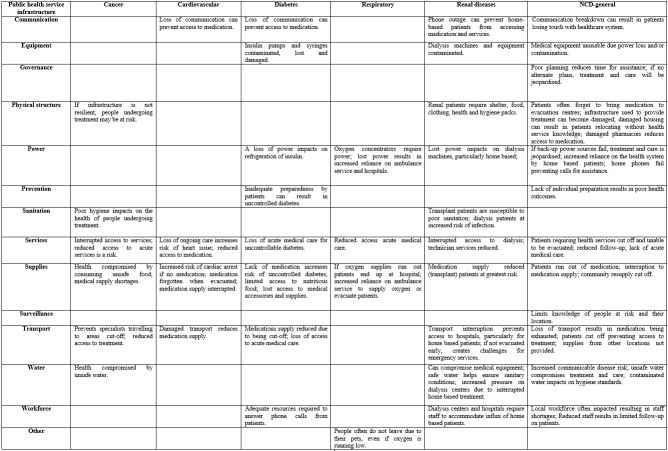
**Table 4.** Reported impact by public health service infrastructure and non-communicable disease - disaster service provider perspective

**** *Communication*

Disrupted communication was reported to impact on people with cardiovascular, diabetes, renal diseases and more generally those with NCDs. The cardiovascular and diabetes impacts relate to a loss in communication preventing access to medication. For people with renal diseases, the risk is predominately for home based patients because damage to phone lines can make it difficult to check if the patient is dialysing. More generally, communication breakdown after a disaster can result in patients being *“…dropped out of the health system…they can’t access health services…we lose them, they’re very hard to re-engage…(I31)”*.

*Equipment*

Equipment damage or interruption can impact people with diabetes, renal diseases and more generally those with NCDs. For people with diabetes equipment can become lost or damaged. Dialysis machines and associated equipment can become contaminated. For example, *“…the machine will clog up…there’s a potential risk of losing the blood in your lines, some stuff leaking through and coming into your blood system, because the machine is not working at 100%...(I3)”*. Also, medical equipment can become unusable for long-periods, particularly due to a reduction in access to technicians and other resources required for repair work.

*Governance*

An interruption to governance can impact on the health of people with NCDs, particularly if there is inadequate planning by agencies and individuals. For example, there are *“…plenty of examples of people with non-communicable diseases needing assistance in shortened time-frames which could have been avoided if they had…that planning and preparation…in place…(I1)”*. Another concern was that *“…people who are at home they tend to be our biggest problem within the district level and the local level… There's no lists and no collective storage of information on these particular people…(FG)”*.

*Physical structure*

Damage to physical structure can impact on the health of people with cancer, renal diseases and more generally NCDs. For people with cancer, curative treatment can become compromised, for example, if* “…someone is in the middle of a curative treatment…you need to be able to have infrastructures to maintain treatment…(I27)”*. Renal patients also require *“…shelter, food, clothing, health and hygiene packs…(I4)”*. More generally, the result is reduced access to treatment and care.

*Power*

An interruption to power can affect people with diabetes, respiratory conditions, renal diseases and more generally those with NCDs. A power outage can impact on refrigeration of insulin for people with diabetes. For people with respiratory and renal diseases there can be a reduction in access to treatment and life-sustaining equipment. Also, for people with respiratory conditions it can result in an increased reliance on the ambulance service, which can have a negative impact on the entire health system. For example,* “…they lose the power; that then impacts on them; their families, ourselves as the ambulance service, because then we've got to either try to supply some oxygen to them in the short term; take them to hospital which then impacts on the hospital's ability to respond to significant events in an emergency department…(I32)”*. A power outage can also impact on communication, preventing calls for assistance.

*Prevention*

Inadequate prevention activities can affect people with diabetes and more generally those with NCDs. Inadequate preparedness by patients can result in uncontrolled diabetes. For example, they *“…tend to have a lot of phone calls mainly in the form of uncontrolled blood sugar… a lot of them will tell you that they lost the paper or the medication…(I26)”*. More generally, a lack of individual preparation for a disaster results in poor health outcomes post-disaster.

*Sanitation*

Damage to sanitation can impact on people with cancer and renal diseases. Poor hygiene can impact the health of people undergoing cancer treatment, for example, *“…when people are on chemotherapy and if their immune system is down…need to make sure that the water quality is goo* *d...”*. Also, for people with renal diseases *“…there’s lots of elements that hygiene’s really essential… I think about their water quality; I think about their exchange areas. Is it clean and hygienic?...(I27)”*. The risk is greatest for transplant patients and those undergoing dialysis treatments.

*Services*

A lack of services can impact people with cancer, cardiovascular, diabetes, respiratory conditions, renal diseases and more generally NCDs. Interrupted access to services and medication can compromise the heath of people with cancer, cardiovascular, diabetes, respiratory conditions and renal diseases. For example,* “…we also had a visiting oncology service. We didn’t actually have a medical oncologist who was located here at Toowoomba Hospital so they were cut off from us…(I7)”*. More generally, follow-up care for people with NCDs can be reduced because patients impacted by a disaster can lose access to treatment and then move without advising the health service.

*Supplies*

Damage to supplies can impact on people with cancer, cardiovascular, diabetes, respiratory conditions, renal diseases and more generally NCDs. For people with cancer this risk relates to unsafe food and medical supply shortages. If medication is not available, people with cardiovascular conditions are at an increased risk of a cardiac arrest, diabetes can become uncontrolled and the overall health of renal transplant patients is at risk. For people with respiratory diseases this can result in an increased reliance on the ambulance service and hospitals to maintain treatment and care. More generally people with NCDs often forget medication when evacuated. For example, *“...if it increases the stress on that person, then there's no doubt it could well have an impact if they have heart disease or cancer or any other condition…(I12)”*.

*Surveillance*

Disruption to surveillance can indirectly impact on people with a range of NCDs. For example, *“…there's a lot of people who do stay at home, who, in fact, may not have been impacted, like their house is okay, but they still might be isolated because of flood roads, so they're the ones we don't know anything about…(I24)”*. Another situation was described where there was* “…no database…(I27)”* to determine *“…how many…patients are home-oxygen dependent or home-dialysis dependent…(I27)”*. The result is an inability to proactively allocate the resources required to help people most at risk of disease exacerbation.

*Transport*

Damage to transport can impact on people with cancer, cardiovascular, respiratory, renal diseases and more generally NCDs. For example,*“…patients from all outlying areas were cut off from…services here at…Hospital…(I7)”. *Also,* “transport mechanisms to get them from home to the treatment facilities, because the majority don't have vehicles…(FG)*”. Overall, the loss of transport reduces access for people with NCDs to services, medication supply and acute medical care.

*Water*

Disrupted water supply can impact on people with cancer, renal diseases and more generally NCDs. Unsafe water can compromise the health of people with cancer and the medical equipment used to treat people with renal diseases. For example, *“The water systems…have a huge impact on the dialysing machines that filter the water…(I3)”* and if not filtered correctly *“…stuff in the water could get into the patient’s blood…(I8)”*. Overall, for people with NCDs there can be an increased risk of infection and communicable diseases due to unsafe water reducing hygiene standards.

*Workforce*

An interruption to the workforce can impact on people with diabetes, renal diseases and more generally NCDs. For example,* “…our biggest thing was staffing…getting staff…(I30)”*. For people with diabetes, a reduction in the workforce can prevent access to specialist advice. For example, after a recent cyclone *“…we had lots of phone calls from patients that they've had high blood glucose levels, unwell, or distressed because they've lost their power, wanting to know how to store medications…(I20)”*. A reduction in staff at dialysis centres can compromise treatment of home based patients who need rapid access to an alternate treatment facility. More generally, a reduction in staff can reduce follow-up on patients.

*Other*

Respondents were aware of cases where people dependent on oxygen (respiratory assistance machines) often do not evacuate due to their pets, even if their supply is running low.

**Mitigation strategies**

Participants described 72 strategies for mitigating the impact of disasters on treatment and care for people with NCDs. The strategies have been categorized by PHI and participant group in Table 5.

**Figure 5:**
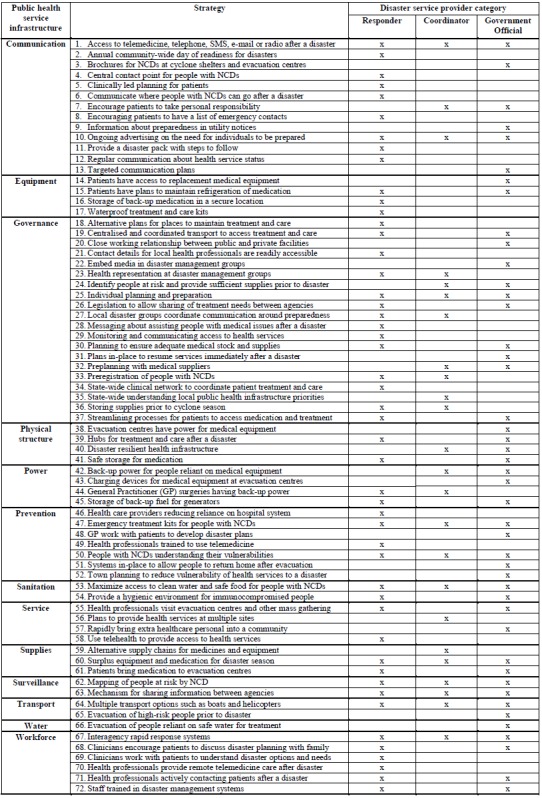
**Table 5.** Mitigation strategies by public health service infrastructure – responsible area

Mitigation strategies were described for all phases of the disaster cycle. For communication, governance, services and the workforce this could include: use of telemedicine; providing brochures at shelters; regular communication about the health service status; close working relationship between public and private sectors; preplanning with medical suppliers; effective town planning; health professionals visiting evacuation centers; and staff trained in disaster management systems. Equipment, physical structure, power, supplies; transport and water strategies include: storage of back-up medication and equipment in secure locations; evacuation centers having power for medical equipment; hubs for treatment and care after a disaster; general practitioner surgeries having back-up power; alternate supply chains; and evacuation of high risk people prior to disaster. Prevention, sanitation and surveillance would include: emergency treatment kits for people with NCDs; people with NCDs understanding their vulnerabilities; providing a hygienic environment; mapping people at risk by NCD; and a mechanism for sharing information between agencies.

The responder group suggested the highest number of mitigation strategies followed by government officials and coordinators. Responders identified 19 unique mitigation strategies, government officials 15 and coordinators three. There were 14 mitigation strategies identified by both responders and government officials. Responders and coordinators identified five similar mitigation strategies. The coordinator and government official groups identified five similar strategies. There were 11 mitigation strategies identified by all groups.

All groups identified mitigation strategies within the PHI categories of communication, governance, prevention, sanitation, supplies, surveillance (described in next paragraph), transport and workforce. Communication related to the need for ongoing advertising on the need for individuals to be prepared and this was complemented by governance where individual planning and preparation is required. From a prevention perspective all groups recommended emergency treatment kits are provided for people with NCDs and for sanitation access to clean water and safe food needs to be maximized. Supplies relate to ensuring a surplus of equipment and medication for the disaster season. For transport multiple options, such as boats and helicopters, are required and this would support the workforce strategy of ensuring inter-agency rapid response systems were in-place.

Two surveillance strategies were recommended by all groups. These included mapping and the sharing of information about people with NCDs. For example, *“…GPs forwarding through…to the Local Disaster Management Group a list of their patients that they believe to be at risk, so that perhaps we can plan to manage those at-risk patients prior to the event rather than wait until after the event…(I27)”*. Another consideration is* “…having a map almost, or an indicator that we’ve got…dialysis patients and these are their addresses. And then you can then work out what you need to from there. This person can last 24 hours without dialysis or two days or two hours…(I6)”*. A common theme from the participants was that having accurate and easily accessible data on people with NCDs would allow disaster service providers to adequately prepare for and respond to a disaster.

## Discussion

Disaster service providers are best placed to lead implementation of strategies for reducing the impact of disasters on people with NCDs. This is because their activities cut across the entire spectrum of the disaster system, including with government, non-government and private sectors. They are employed in communities where disasters occur and the core elements of their work are directly linked to enhancing PHI resilience. Specific measures include: increasing the use of telemedicine; preplanning with medical suppliers; effective town planning; health professionals visiting evacuation centers; evacuation centers having power for medical equipment; hubs for treatment and care after a disaster; evacuation of high risk people prior to disaster; mapping people at risk by NCD; and a mechanism for sharing information between agencies.

Within the disaster service provider group, responders are best placed to lead and guide implementation of the mitigation strategies. For example, the *“…local system needs to feed into the hierarchy of command and the patient should only be having contact with that local person…(I4)”*. This could only occur with support from coordinators and government officials. The importance of responders leading this work is further supported by the high number of mitigation strategies recommended by this group, which reflects their work ‘on the ground’ before, during and after disasters. Meanwhile, the coordinator group provides the much needed conduit between responders and government officials.

Disaster service providers consider themselves responsible for maximising treatment and care for people with NCDs. For example, a situation was described where *“…we knew the bridge was going to go under, so we ensured that the people that needed medication were identified in that township and we ensured that they had their medications, because they were going to be isolated…(I9)”*. This combined with the wide range of disciplines among disaster service providers demonstrates they can provide the leadership required to systematically expand the focus of disaster systems to include NCDs. To guide implementation of this change PHI, services and NCD considerations are discussed in the following along with implementation of the mitigation strategies outlined in Table 5.

**Public health service infrastructure (PHI) considerations**

A relationship was identified between damage to PHI and an increased risk of disaster exacerbated NCDs. This concern is consistent with the literature which has found that when PHI are destroyed or rendered inaccessible access to chronic care treatment and medication is jeopardized.^19^^,^ 55 Also, acute care can also become compromised and this presents a risk for people with NCDs, for example, orthopedic surgery is at much higher risk when a patient has poorly controlled cardiovascular disease.^57^

An interruption to transport will not only decrease access to services but reduce the ability to provide care. To overcome this risk, strategies need to be in-place to allow the disaster service providers to provide care and treatment during and immediately after a disaster.^30^ This could include disaster and health planners encouraging those who provide care to have personal disaster plans. This proposal complements other research which has found the willingness of nursing staff to attend a workplace after a disaster increases by eight times if they have a disaster plan.^58^ Also, people are more likely to attend work after a disaster if their colleagues are prepared for disaster response.^59^

Pets should be considered part of PHI. For example, people *“…won’t leave because of their pets…(I10)”* even if *“…their oxygen was running really low…(I10)”*. There is also the risk that people with pets will not attend evacuation centres and there can be difficulty in convincing farmers to evacuate when livestock is at risk.^15^ For this reason, when developing plans it is important disaster and health planners consider how pets will be handled.

The identification of additional descriptors (Table 1) for the PHI themes identified by Ryan et al^30^ is another step towards describing and categorizing PHI priorities for disaster management systems. The next step is a validation of the PHI themes, descriptors and priorities. The result would be a clearer understanding of PHI before, during, and after a disaster. This information will be vital to systematically inform preparation, response, and recovery activities and plans for reducing the risk of disaster exacerbated NCDs through PHI resilience.

**Non-communicable disease considerations**

The level of attention given to disaster risk reduction activities in mitigating NCDs needs to be accelerated, particularly due to global population aging, increasing obesity and overweight and decreasing physical activity.^11^^,^ 12 ^,^ 13 ^,^ 14 The challenge will be to broaden the focus of disaster management activities for health from response and recovery within the disaster cycle model to a more proactive approach which emphasizes prevention and mitigation.^60^ For example, participants described an option that *“…I don't think there's very good pre-planning for an event in relation to those people that do have chronic diseases…(I32)”*. Addressing this will require the health system to increase capacities and relationships across community, non-government, government and service provider sectors (including electricity and telephone companies).^60^ The Sendai Framework statement concerning chronic diseases (NCDs) provides the platform for this to occur.^8^

Another consideration beyond the NCDs targeted by this research is the impact disasters may have on drug users and people in drug addiction treatment programs. Research participants discussed the need to consider the impact a breakdown of services or supplies may have on people reliant on methadone and other treatments for drug addiction. Also, evacuation centers and shelters are often ill equipped to address acute drug withdrawals and maintain specialized services for treatment programs.^61^ The result is an over reliance on emergency room dosing that lead to unsafe or suboptimal dosing.^62^ This issue could be addressed by developing guidance to rapidly verify identification, establish proof of treatment and ascertaining dosage information.^61^ By recognizing this challenge, strategies can be developed to ensure drug users and people in drug addiction treatment programs receive the care needed.

**Mitigation strategies – implementation**

The mitigation strategies presented in Table 5 provide the theoretical basis required for demonstrating how PHI resilience can maximize the treatment and care available for people with NCDs before, during and after a disaster. Although the responder group proposed more strategies than the governmental officials and coordinators, it is the entire set of strategies that provides the theoretical basis for addressing the problem. The mitigation strategies are interrelated via PHI because if one component fails the health and well-being of people with NCDs will be compromised.

To sustainably implement the mitigation strategies they will need to become integrated into effective performance and monitoring of the disaster response and health sector during non-disaster periods. For this to occur, the strategies should be integrated into business and strategic plans. This could include, for example, identifying a disaster resilient location for back-up treatment and care and working with primary health care facilities to design a hub model of care post-disaster. This approach would ensure PHI disaster resilience, and the benefits this will have for the ongoing care of people with NCDs, is integrated into system-wide policies, plans, programmes and budgets.^8^

While sharing of information across agencies about people with NCDs has been identified as a strategy there are ethical and community willingness considerations. For example, in Australia vulnerable persons registers (generally include people with NCDs) are maintained at the local government level and registration is voluntary.^63^ The result is often an underestimate of the number of people at risk, for example, in the State of Victoria, Australia, there are less than 1,350 registered as vulnerable out of a population of just under six million.^64^^,^ 65 To help address this problem, it is recommended an integrated system be developed that allows local healthcare providers to provide and update information on patients they believe are vulnerable to disaster managers at local government levels. This would occur with permission from the patient.

A collaborative governance approach is required to implement the mitigation strategies due to the wide range of disciplines, organizations and sectors involved.^66^ Such an approach would be an amalgamation of organizations beyond the current disaster management system (government agency focus) to allow community and private organizations such as universities, primary health care sector (general partitioners and pharmacies) and transport companies to help solve this problem.^67^ The process would include: engaging in comprehensive and shared planning; formal communication across multiple levels; and pooling and jointly acquiring resources to implement the mitigation strategies.^68^ This approach would complement the comprehensive, all hazards, all agencies approach to disaster management in Queensland, Australia, and reflect the principles of intersectoral collaboration.^68^^,^ 69 ^,^ 70

Finally, the research has presented theoretical strategies for reducing the impact of disasters on the health and well-being of people with NCDs through PHI resilience. Further research needs to be completed to test the effectiveness of the strategies presented. For example, will strategies make a difference by NCD, location and disaster type. This would include working with disaster service providers, primary health care sector, community based organizations and people with NCDs to rank and prioritize the strategies. The result would be strategies which have clearly defined roles, responsibilities and options for enhancing PHI resilience. Ultimately, this will help implement the Sendai Framework and, most importantly, result in sustainable strategies that protect the health of people with NCDs before, during and after a disaster.

## Limitations

The research direction was informed by the authors professional and research experience in the public health aspects of disasters in Australia at local, state, national and international levels. The lead author has also been part of preparedness and response activities to cyclones that have impacted some of the areas subject to the research. To address this limitation, the second author assisted with the research design and verified the data.

The research was limited to disaster service providers in Queensland, Australia, who have predominately prepared for, and responded to, cyclones, floods and storms in Australia. This group has a vital role in influencing the impact of disasters on PHI and maximizing treatment and care for people with NCDs. This type of role is similar in other parts for the world. For this reason, it is proposed the findings can be transferred to the same disaster types across the world where there are similar disease burdens/trends, however, caution is urged as all systems are unique.

Another potential limitation is that the research was limited to a high-income country setting. Therefore, caution should be taken to applying the findings to low and middle income countries, particularly as the PHI and NCD priorities and challenges may differ.

## Conclusion

Disaster service providers can play a vital role in reducing the risk of disaster exacerbated NCDs through PHI resilience. They are often employed in communities where disasters occur and the core elements of their work are directly linked to enhancing PHI resilience to maximize treatment and care at a local level. This combined with the wide range of disciplines demonstrates they can provide the leadership required to systematically expand the focus of disaster systems to include NCDs. Specific measures include: increasing the use of telemedicine; preplanning with medical suppliers; effective town planning; health professionals visiting evacuation centers; evacuation centers having power for medical equipment; hubs for treatment and care after a disaster; evacuation of high risk people prior to disaster; mapping people at risk by NCD; and a mechanism for sharing information between agencies. To sustainably implement the mitigation strategies they will need to become integrated into effective performance and monitoring of the disaster response and health sector during non-disaster periods. For this to occur, the strategies should be integrated into business and strategic plans. Achieving this will help implement the Sendia Framework and, most importantly, result in sustainable strategies that protect the health of people with NCDs before, during and after a disaster.

## Data Availability

The raw data used for this analysis can be accessed via https://dx.doi.org/10.6084/m9.figshare.4127064. All demographic data have been removed to ensure all respondents were appropriately de-identified. Due to ethical restrictions, requests for the interview and focus group transcripts will only be considered upon request. For further information regarding data availability please contact Benjamin Ryan at benjamin.ryan@my.jcu.edu.au.

## Corresponding Author

Benjamin Ryan, PhD Candidate, MPH, BScEH

1 James Cook Drive, Townsville QLD 4811, Australia

E-mail: benjamin.ryan@my.jcu.edu.au

## Competing Interests

The authors have declared that no competing interests exist.
